# A systematic review on the association of sleep-disordered breathing with cardiovascular pathology in adults

**DOI:** 10.1038/s41533-022-00307-6

**Published:** 2022-10-17

**Authors:** Anna Khokhrina, Elena Andreeva, Jean-Marie Degryse

**Affiliations:** 1 Institute for Health and Society, Université Catholique de Louvain, IRSS, Clos Chapelle-aux-Champs, 30/10.15, 1200 Brussels, Belgium; 2 Northern State Medical University, av. Troitsky, 51, 163000 Arkhangelsk, Russia; 3 Department of Public Health and Primary Health Care, K.U. Leuven, Kapucijnenvoer 33, B3000 Leuven, Belgium

**Keywords:** Epidemiology, Respiratory tract diseases

## Abstract

Sleep-disordered breathing (SDB) is characterized by repeated breathing pauses during sleep. The prevalence of SDB varies widely between studies. Some longitudinal studies have found an association of SDB with incident or recurrent cardiovascular events. We sought to systematically describe the current data on the correlation between SDB and cardiovascular pathology. Studies were included if they were original observational population-based studies in adults with clearly diagnosed SDB. The primary outcomes include all types of cardiovascular pathology. We carried out pooled analyses using a random effects model. Our systematic review was performed according to the PRISMA and MOOSE guidelines for systematic reviews and was registered with PROSPERO. In total, 2652 articles were detected in the databases, of which 76 articles were chosen for full-text review. Fourteen studies were focused on samples of an unselected population, and 8 studies were focused on a group of persons at risk for SDB. In 5 studies, the incidence of cardiovascular pathology in the population with SDB was examined. In total, 49 studies described SDB in patients with cardiovascular pathology. We found an association between SDB and prevalent /incident cardiovascular disease (pooled OR 1.76; 95% CI 1.38–2.26), and pooled HR (95% CI 1.78; 95% CI 1.34–2.45). Notably, in patients with existing SDB, the risk of new adverse cardiovascular events was high. However, the relationship between cardiovascular disease and SDB is likely to be bidirectional. Thus, more large-scale studies are needed to better understand this association and to decide whether screening for possible SDB in cardiovascular patients is reasonable and clinically significant.

## Introduction

Sleep-disordered breathing (SDB) is characterized by repeated breathing pauses during sleep^1^. According to the American Academy of Sleep Medicine (AASM), the main types of respiratory events are obstructive apnea or hypopnea, central apnea and mixed apnea^2^. A respiratory event is scored as an apneic episode if it meets the following criteria: a drop of the oronasal flow ≥90%, the duration of the event is over 10 s, and it is associated with continued inspiratory effort^2^. In obstructive hypopnea, the duration of the ≥30% drop in oronasal flow lasts ≥10 s and is associated with ≥3% oxygen desaturation from a pre-event baseline or with an arousal^2^. The event is defined as central if it meets apnea criteria and is associated with a lack of inspiratory effort^2^. If the event meets the apnea criteria and is associated with a lack of inspiratory effort at the beginning of the event, followed by resumption of inspiratory effort in the second portion of the event, it is classified as mixed apnea^2^. In the AASM guidelines, there are three additional types of respiratory events that are less prevalent: respiratory effort-related arousals (RERA), hypoventilation and periodic breathing. SDB is labeled as mild, moderate or severe based on the number of apnea and/or hypopnea episodes per hour of sleep, known as the apnea-hypopnea index (AHI)^3^. The prevalence of SDB varies widely because of the heterogeneity of the study methodology, different diagnostic standards (full polysomnography or portable sleep monitoring) and different AHI cut-offs. The prevalence of SDB has been reported to range between 17 and 49% for AHI ≥ 5^4,5^ and between 4.6 and 43.7% for AHI ≥ 15^6,7^.

Some longitudinal studies have found an association of SDB with incident or recurrent cardiovascular events: heart failure, myocardial infarction, stroke/transient ischemic attack^8–12^. Previous studies^7,13,14^ have shown that there is a dose-response relationship between the severity of SDB and the risk of various clinical manifestations of cardiovascular disease (CVD). These studies were conducted predominantly in cardiology patients or patients with an established diagnosis of SDB. Only a few studies focusing on this association in an unselected population have been published^14–16^.

SDB influences CVD through numerous pathophysiological mechanisms. The most likely causal pathway through which SDB causes CVD is thought to be intermittent hypoxia, endothelial dysfunction and inflammation, repetitive arousal from sleep, large intrathoracic pressure variations and increased sleeping blood pressure^17–19^. While the acute, unfavorable effects of sleep disorders on cardiovascular physiology have been well characterized, less research is available on how strong the effect of SDB is on symptomatic CVD. Previous systematic reviews were mostly based on a particular type of cardiovascular pathology or cardiovascular outcome^20–22^.

The main objective of this systematic review is to systemize the current data on the association between SDB and cardiovascular pathology.

## Methods

### Search question and search strategy

Our systematic review was performed according to the PRISMA and MOOSE guidelines for systematic reviews^23,24^. A systematic literature review was added to the PROSPERO register (registration number is CRD42018082314).

A ‘PACO’ (Patient-Type of Association-Comparison-Outcome) analogous to the ‘PICO’ (Patient-Intervention-Comparison-Outcome) for systematic reviews of interventional studies, was created to guide our systematic review of observational studies.

Our basic aim was to review the association between SBD and prevalent and/or incident CVD. Our research question was further operationalised as: (P) Patients with a documented SBD (A): cross-sectional or longitudinal associations (C) Persons without SBD, (O) Prevalent/incident CVD.

Since we aimed to investigating the association between SBD and CVD in a bidirectional way, we decided to search for complementary studies that investigated the prevalence and/or incidence of SBD in patients with an established CVD.

The search was made in PubMed, Ovid and EMBASE databases in February 2022 without limitations on language, publication year or country. The search strategy included only terms relating to or describing the association of SDB and cardiovascular pathology. The search terms were adapted for use with bibliographic databases separately and in combination with database-specific filters.

The search terms were obstructive sleep apnea syndrome, obstructive sleep apnea, sleep apnea, obstructive sleep apnea hypopnea syndrome, sleep-disordered breathing, heart failure, cardiac insufficiency, heart insufficiency, atrial fibrillation, cerebral vascular accident, stroke and myocardial infarction.

The identified articles were screened by title and abstract and selected for full text review if they met the following inclusion criteria developed according to the objectives of the review.

1. Original observational (including cross-sectional, cohort and case–control studies) that were population-based and concerned the adult population.
2. Studies in hospital cardiovascular departments, in sleep centers and studies of unselected populations.
3. The diagnosis of SDB was based on the apnea-hypopnea index or respiratory disturbance index (RDI), which were obtained by the gold standard for diagnosing full polysomnography or portable home sleep apnea testing^2^.
4. The primary outcome concerned the following types of cardiovascular pathology: heart failure, cardiovascular disease, coronary heart disease, atrial fibrillation, stroke, myocardial infarction.
5. A diagnosis of cardiovascular pathology was based on the clinical evidence, laboratory data and/or functional methods of examination.

Exclusion criteria.

1. Studies on children and pregnant women.
2. The diagnosis of SDB was based on a questionnaire or ICD-9 diagnosis code.
3. If only risk factors for CVD were studied as an outcome.

### Data extraction

Titles and/or abstracts of studies retrieved by the search strategy were screened independently by two reviewers (AH and EA) to identify studies that potentially met the inclusion criteria. The full text versions of these potentially eligible studies were retrieved and independently assessed for eligibility by two review team members. Any disagreement between them over the eligibility of studies was resolved through discussion with a third reviewer (JD). A standardized form was used to extract data from the included studies for assessment of study quality and evidence synthesis. Extracted information included study setting; study population and participant demographics and baseline characteristics; study design and methodology; type of SDB and cardiovascular pathology; observation time, if possible; and results (prevalence in percentage, odds ratios and hazard ratios, if possible). Two reviewers extracted data independently; discrepancies were identified and resolved through discussion (with a third author when necessary). Missing data were requested from study authors.

### Quality assessment

Two reviewers (AH and EA) independently assessed the methodological quality of the selected studies with the Quality Assessment Tool for Case-Control, Cohort and Cross-sectional Studies, depending on the study design (https://www.nhlbi.nih.gov/health-topics/study-quality-assessment-tools).

### Statistical analysis

We looked at data from cross-sectional studies and cohort studies separately to estimate the effect of SDB on the risk of cardiovascular pathology. We collected multivariable-adjusted (if possible) hazard ratios (HR) or odds ratios (OR) from the original studies. When a study did not report the OR of the outcomes of interest, we calculated an unadjusted OR based on the original data of events. We performed random effects analysis to quantify the dispersion of effect sizes in a meta-analysis. We pooled the HR and OR separately with a 95% confidence interval (CI) for adverse cardiovascular outcomes within a random effects model (DerSimonian-Laird) that incorporates between-study heterogeneity. The Cochran Q test (at a significance level of *p* < 0.10) and the I^2^ statistic were used to examine statistical heterogeneity across studies. We planned to assess publication bias using visual evaluation of the funnel plots. All analyses were performed with Cochrane Review Manager software (version 5.3).

### Reporting summary

Further information on research design is available in the Nature Research Reporting Summary linked to this article.

## Results

### Study selection and characteristics

Using the above-mentioned search terms, 2681 articles were selected from the PubMed, Ovid and EMBASE databases after deduplication. We excluded 2535 articles for the following reasons: did not relate to the correlation of SDB and cardiovascular pathology (*n* = 2516); were literature reviews (*n* = 17) and were systematic reviews (*n* = 2). One hundred forty-six articles were eligible for full text evaluation. Of these articles, 7 were not diagnostic, 1 was performed on a very select population (combination of atrial fibrillation and erectile dysfunction), 3 were on patients with both CVD and SDB, in 23 the diagnostic standard for SDB was not used, in 27 there were no clinical diagnoses of CVD made, and 7 did not contain full texts. In 86 out the 2681 initially screened studies disagreement between reviewers appeared around their eligibility; After discussion with a third reviewer 2534 were excluded. After reviewing the full text articles no further disagreement emerged. Seventy-nine articles were eligible based on the aforementioned criteria. Some studies were excluded for more than one reason.

**Fig. 1:**
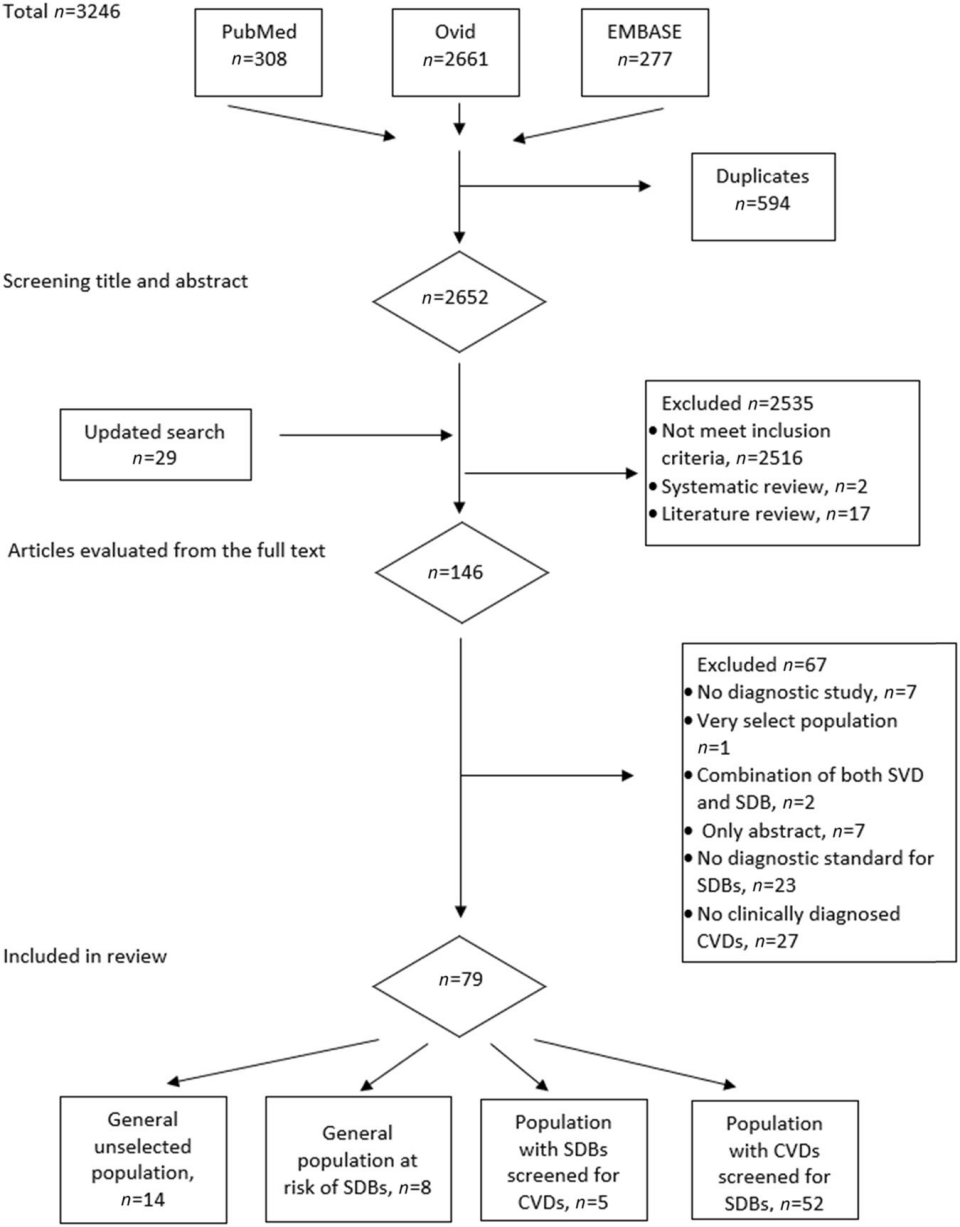
Flow chart of the study selection process for meta-analysis. SDB sleep-disordered breathing, CVD cardiovascular disease.

Fourteen studies were performed on samples of an unselected population, and eight were performed on a group of persons at risk for SDB. In five studies, the incidence of cardiovascular pathology in the population with SDB was examined. Forty-nine studies described SDB in patients with cardiovascular pathology. The flow chart of the literature search is shown in Fig. 1. A total 38 studies were cross-sectional, 33 studies were cohort studies, and 5 studies were case-controls. The characteristics of the studies grouped by type of population are listed in Tables 1 to 5.

**Table 1:** Association of sleep-disordered breathing and cardiovascular disease in an unselected population: study characteristics, outcomes and results.

First author	Year	Country	Study Design	Sample Size	Age	Diagnostic Standard	Outcome	Follow-up Period	AHI cut-off		Overall prevalence	Odds ratio (95% CI)	Hazard ratio (95% CI)	Adjusted for
Roca G.Q. et al.	2015	USA	cohort	1645	62.5 ± 5.5	PSG	All-cause mortality, incident CHD and HF	13.6 ± 3.2 years	15		Men 23%	–	–	Age, BMI, prevalent hypertension and diabetes, systolic BP, smoking status, and use of medications.
											Women 10.4%	OR: 1.25 (1.02–1.52)	HR: 1.33 (1.03–1.74)	
Javaheri S. et al.	2015	USA	cohort	2865	76.3 ± 5.5	PSG	Incident HF	7.3 years	AHI > 15		43.7%	OR: 1.9 (1.4–2.6)	–	Clinic, age, race, BMI, history CAD, HF, stroke, diabetes, hypertension, smoking, alcohol use, and physical activity.
									CAI > 5		7.3%	OR: 2.16 (1.4–3.4)	HR: 1.79 (1.16–2.77)	
Redline S. et al.	2010	USA	cohort	5422	median 72	PSG	Incident stroke (nonfatal or fatal)	8.7 years	15		20.2%	OR: 2.26 (1.45–3.52)	HR: 2.64 (1.01–6.88)	Age, BMI, smoking status, systolic BP, use of medications, diabetes status, and race.
Gottlieb D.J. et al.	2010	USA	cohort	4422	64 (57, 72) men	PSG	CHD, HF	Median of 8.7 years	15		24% men	–	CHD HR: 1.10 (1.00–1.21) HF HR: 1.13 (1.02–1.26)	Age, race, BMI, smoking, total and HDL cholesterol, lipid- lowering meds, diabetes mellitus, systolic BP, diastolic BP, and anti- hypertensive medications.
					66 (58, 74) women						11% women			
Munoz R. et al.	2007	Spain	cohort	394	median 77, 28 years	PSG	Ischemic stroke	6 years	30		24.1%	–	HR: 2.52 (1.04–6.01), P 0.04	Sex
Marshall N.S. et al.	2014	Australia	cohort	400	55.1 ± 8.2	PSM	All-cause mortality (CVD, CHD, Stroke)	20 years	5		20.6%	–	HR: 0.5 (0.27–0.99)	Age, sex, BMI, smoking status, total cholesterol, HDL cholesterol, mean arterial pressure, diabetes, angina, and history of CVD.
									15		4.6%		HR: 4.2 (1.9–9.2)	
Stone K.L. et al.	2015	USA	cohort	2872	76.3 ± 5.5	PSG	Incident stroke	7.3 years	5		–	–	HR: 1.83 (1.12–2.98)	Age, clinic, race, BMI, and smoking
Munoz R. et al.	2012	Spain	cohort	394	median 77.3	PSG	Ischemic stroke	4.5 years	CAI > 1		–	–	HR: 2.65 (1.08–6.49)	AF
									CAI > 3				HR: 3.08)1.26–7.52)	AF and sex.
Hla Khin Mae et al.	2015	USA	cohort	1280	47 ± 8	PSG	CHD, HF	24 years	5–15		14%	_	HR: 1.9 (1.05–3.5)	Age, sex, BMI, and smoking.
									15–30		5%		HR: 1.8 (0.85–4.0)	
									>30		4%		HR: 2.6 (1.1–6.1)	
May A. M. et al.	2016	USA	cohort	843	75 ± 5	PSG	Incident AF	65 ± 0.7 years	CSA > 5		6%	OR: 9.97 (2.72–36.50)	_	Age, clinic, race, BMI, history of CVD, hypertension, diabetes, stroke, COPD, pacemaker placement, total cholesterol, use of medications, and alcohol use.
									AHI > 15		41,7%	OR: 2.64 (1.16–6.00)		
Tung P. et al.	2017	USA	cohort	2912	62.8 ± 11.2	PSG	Incident AF	5.3 years	AHI	5	49%	–	_	Age, clinic, race, BMI, history of CVD, hypertension, diabetes mellitus, stroke, COPD, pacemaker placement, total cholesterol, use of cardiovascular medications, and alcohol.
										15	19%	–		
										30	7%	–		
									CAI > 5		2.5%	OR: 3.00 (1.40–6.44)		
Kwon Y. et al.	2015	USA	cross-sectional	2048	68.4 ± 9.2	PSG	AF	–	15		33.74%	OR: 1.23 (1.01–1.50)	_	Age, field center, race/ethnicity, sex, BMI, height, smoking status, diabetes, systolic BP, and medications.
Arzt M. et al.	2005	USA	cross-sectional	1475	47 ± 8	PSG	Stroke	–	5		17%		–	
									20		7%	OR: 3.83 (1.17–12.56)		Age, sex, BMI, alcohol, smoking, diabetes, and hypertension.
Cho E.R. et al.	2013	Korea	cross-sectional	746	59.3 ± 7.2	PSG	Cerebral infarction	–	15		12.06%	SCI OR: 2.44 (1.03–5.80)	–	Age, hypertension and diabetes mellitus.
												Lacunar infarction OR: 3.48 (1.31–9.23)		

*AF* atrial fibrillation, *AHI* apnea-hypopnea index, *BMI* body mass index, *BP* blood pressure, *CAD* coronary artery disease, *CAI* central apnea index, *CHD* coronary heart disease, *COPD* chronic obstructive pulmonary disease, *CVD* cardiovascular disease, *HDL* high density lipoprotein, *HF* heart failure, *HR* hazard ratio, *OR* odds ratio, *PSM* portable sleep monitor, *PSG* polysomnography, *SCI* silent cerebral infarction.

**Table 2:** Association of sleep-disordered breathing and cardiovascular disease in a population at risk of SDB: study characteristics, outcomes and results.

Author	Year	Country	Study Design	Sample Size	Age	Outcome	Follow-up period	AHI cut-off	Prevalence	OR (95% CI)	HR (95% CI)	Adjusted for
Cadby G. et al.	2015	Australia	cohort	6841	48.3 ± 12.5	AF	11.9 years	5	63.6%	OR: 2.8 (2.2–3.6)	HR: 1.55 (1.21–2.00)	Age, sex, height, BMI, hypertension, valvular disease, stroke/TIA, coronary or peripheral artery disease, COPD, chronic renal disease, HF, and diabetes.
Gami A.S. et al.	2013	USA	cohort	10,701	53 ± 14	Sudden cardiac death	5.3 years	5	78%	–	–	
								20	–		HR 1.05 (1.00–1.09)	Univariate analyses
Shah N.A. et al.	2010	USA	cohort	1436	60	CVD	2.8 years	5	71%	–	HR: 2.06 (1.10–3.86)	Age, race, sex, smoking, alcohol use, BMI, AF, hypertension, hyperlipidemia, and diabetes.
Yaggi H. K. et al.	2005	USA	cohort	1022	60,9	Incident stroke/TIA or all-cause death	3.4 years	5	68%	–	HR: 1.97 (1.12–3.48)	Age, sex, race, smoking, alcohol use, BMI, presence of diabetes mellitus, hyperlipidemia, AF, and hypertension.
Gami A.S. et al.	2007	USA	cohort	3542	49 ± 14	Incident AF	4.7 years	5	74%	–	HR: 2.18 (1.34–3.54)	Univariate analyses
Kendzerska T. et al.	2014	Canada	cohort	10,149	49,9 ± 14,1	MI, stroke, CHF, revascularization procedure, all-cause death	68 months	5	79.2%	–	HR: 1.12 (1.05–1.2)	BMI, age, sex, smoking, hypertension, diabetes, MI, stroke, and HF.
Selim B.J. et al.	2016	USA	cross-sectional	697	58.7 ± 12.1	Nocturnal cardiac arrhythmias	–	5	77%	–	–	
								15	56%	OR: 2.24 (1.48–3.39)		Age, BMI, sex, and CVD.
Roche F. et al.	2002	Switzerland	cross-sectional	147	54.5 ± 10.7	Nocturnal paroxysmal asystole	0	10	44.9%	OR: 9.5 (1.14–79.2)	–	Not adjusted

*AF* atrial fibrillation, *AHI* apnea-hypopnea index, *BMI* body mass index, *COPD* chronic obstructive pulmonary disease, *CVD* cardiovascular disease, *HF* heart failure, *HR* hazard ratio, *MI* myocardial infarction, *OR* odds ratio, *PSM* portable sleep monitor, *PSG* polysomnography, *TIA* transient ischemic attack.

**Table 3:** Association of sleep-disordered breathing and cardiovascular disease in patients with an established cardiovascular pathology.

First Author	Year	Country	Sample Size	Age	Diagnostic standard	Type of CVD	Outcome	Follow-up period	AHI cut-off	Prevalence		Odds ratio (95% CI)	Hazard ratio (95% CI)	Adjusted for
Sano K. et al.	2013	Japan	178	71.4 ± 1.3	PSG	CHF	Death from CV causes (worsening HF, ventricular tachyarrhythmia systemic embolism, stroke, acute MI, or aortic dissection)	22 months	CAI >7.5	38.7%		AF OR: 1.03 (1.02–2.51)	HR: 1.29 (1.16–2.32)	For OR: age, sex, BMI, NYHA class, LVEF, brain natriuretic peptide, CAI, minimum SpO2, duration of SpO2 <90%, C-reactive protein, and the use of a beta-blocker. For HR: age, NYHA class, LVEF, C-reactive protein, brain natriuretic peptide, and minimum SpO2.
												Sinus pause OR: 1.12 (1.08–1.35)		
												Nonsustained ventricular tachycardia daytime OR: 1.22 (1.00–6.92), nighttime OR: 3.57 (1.06–13.1)		
Mooe T. et al.	2001	Sweden	408	< 70 years	PSG	CAD	Composite of death, CV events, and MI	5.1 years	≥10	34%		composite end point OR: 1.62 (1.09–2.41)	HR: 2.98 (1.43–6.20)	Age, sex, BMI, and hypertension.
												CV events OR: 3.41 (1.73–6.71)		
Silvia L. et al.	2016	Portugal	73	63.5 ± 10.3	PSM	Acute Coronary Syndrome	All-cause mortality, MI, and myocardial revascularization	75 months	>5	63%		–	–	Sex
									≥15	–			HR: 3.58 (1.09 −17.73)	
Shah R.V. et al.	2014	USA	403	median 57	PSG	AF	All-cause mortality/HF hospitalization	3.3 ± 0.5 years	>5	19%		–	HR: 2.14 (1.16–3.98)	Age, male sex, BMI, history of HF, hyperlipidemia, hypertension, diabetes, left ventricle mass-to-volume ratio, left ventricular end-systolic volume index, left ventricular myocardial infarction, and right ventricular ejection fraction.
Ponsaing L.B. et al.	2017	Denmark	63	median 67.5	PSG	Stroke/ TIA	Mortality	19–37 months	>24	–		–	stroke severity HR: 10.95 (1.25–95.14	Age, disability measured with the modified Barthel index, and atrial fibrillation were nonsignificant.
													disability HR: 11.08 (1.23–99.52)	
Emdin M. et al.	2017	Italy	525	66 ± 12	PSM	Systolic HF	Cardiac mortality	median 34 month	>5	CSA 38.2%	nighttime 49.9%	–	HR: 1.02 (1.01–1.04)	Age, N-terminal pro–B-type natriuretic peptide, estimated glomerular filtration rate, and LVEF.
											daytime 28.4%		HR: 1.03 (1.01–1.06)	
										OSA 4.5%	nighttime 9%		HR: 1.02 (1.01–1.04)	
											daytime 1.5%			
Lee Chi-Hang et al.	2011	Singapore	120	52.7 ± 9.8	PSM	Acute MI	Death, reinfarction, stroke, unplanned target vessel revascularization, and HF requiring hospitalization.	18 months	<30	58%		–	–	Age, and BMI.
									≥30	42%			HR: 5.36 (1.01–28.53)	
Javaheri S. et al.	2010	USA	30,719	67.1 ± 12.1	PSG	Chronic HF	Incidence, treatment, outcomes, and economic cost of sleep apnea in new-onset HF.	2-year survival rate	>5	97%		–	HF HR: 0.33 (0.21–0.51)	Age, sex, and Comorbidities.
Khayat R. et al.	2014	USA	1117	CSA 60.3 ± 14.7 OSA 60.3 ± 13.0	PSM	Acute HF	Mortality	3 years	>15	CSA − 31%		–	HR: 1.61 (1.1–2.4)	LVEF, age, BMI, sex, race, creatinine, diabetes, type of cardiomyopathy, CAD, chronic kidney disease, discharge systolic BP < 110, hypertension, discharge medications, initial length of stay, admission sodium, hemoglobin, and blood urea nitrogen.
										OSA − 47%			HR: 1.53 (1.1–2.2)	
O. Parra A. et al.	2004	Spain	161	72 ± 9	PSM	Stroke/ TIA	Death and time of survival since the neurological event.	22.8 months	>10	72%		–	HR: 1.05 (1.01–0.08)	Age, middle cerebral artery involvement, and coronary disease.
									>20	47.2%				
									>30	28%				
									>40	11.2%				
									>50	5%				
Hang L.C. et al.	2016	Singapore, China, Brazil, India, Myanmar	1311	58.2 ± 10.3	PSM	Percutaneous coronary intervention	MACCEs, secondary end points: all-cause mortality, target vessel revascula-rization, stent thrombosis, and hospitalization for HF.	1.9 years	≥15	45.3%		–	HR: 1.57 (1.10–2.24)	Age, sex, ethnicity, BMI, diabetes mellitus, and hypertension.
Bolotova M.N. et al.	2008	Russia	120	57.5 ± 1.2	PSG	HPT	Death stroke, MI, HF, and AF.	4.1 years	>15	53%		Stroke OR: 1 (0.32–11)	–	Not adjusted
												MI OR: 0.56 (0.17–1.42)		
												HF OR: 1.44 (0.36–1.33)		
												AF OR: 1.54 (0.63–3.79)		
Uchoa C. et al.	2015	Brazil	67	58 ± 8	PSG	CAD	MACCEs, Secondary end points (individual MACCEs, typical angina, and arrhythmias).	4.5 years	>15	56%		MASSE OR: 4.10 (1.94–385.24)	–	Age, Male sex, waist circumference, statins, angiotensin-converting enzyme inhibitor, angiotensin receptor blocker, and LVEF.
												New vascularization OR: 2.02 (1.21–64.22)		
												Typical angina OR: 10.05 (1.12–62.25)		
												Atrial fibrillation OR: 12.56 (1.44–159.21)		
Szymanski F.M. et al.	2015	Poland	251	57.6 ± 10	PSM	AF	Reoccurrence of the AF	30 months	>5	45.4%		OR: 2.58 (1.52–4.38)	–	Multivariate logistic regression analysis.
Zhao Liang-Ping et al.	2015	Singapore	41	52.2 ± 9.6	PSM	Acute MI	Cardiac death, nonfatal MI, hospitalization for angina and/or congestive HF.	5 years	>5	34.1%	15-30 22.0%	OR: 1.044 (1.003–1.086)	–	–
											>30 34.1%			
Fan Jingyoa et al.	2019	China	804	57 ± 10.2	PSM	Acute CoronarySyndrome	MACCE	1 Year	>15	50.1%			HR: 1.55 [0.94–2.57] after 1 Year HR: 3.87 [1.20–12.46]	Age, sex, BMI, HT, diabetes, PCI procedure and minimum oxygen saturation.
Yuhui Huang et al.	2020	China	382	No Osa: 51 ± 16 Osa: 57±	PSM	Decompensated HF	Death, heart transplantation or implantation of LVAD. Unplanned hospitalization for worsening HF, ACS, significant arrhythmias, Stroke	19.7 months	>15	49.5%			HR: 1.14 [0.859–1.532]	No

*AF* atrial fibrillation, *AHI* apnea-hypopnea index, *BMI* body mass index, *BP* blood pressure, *CAD* coronary artery disease, *CAI* central apnea index, *CHF* congestive heart failure, *CV* cardiovascular, *CSA* central sleep apnea, *HF* heart failure, *HPT* hypertension, *HR* hazard ratio, *LVEF* left ventricle ejection fraction, *MACCEs* major adverse cardiac and cerebrovascular events, *MI* myocardial infarction, *OSA* obstructive sleep apnea, *OR* odds ratio, *PSM* portable sleep monitor, *PSG* polysomnography.

Cohort studies: characteristics, outcomes and results.

**Table 4:** Association of sleep-disordered breathing and cardiovascular disease in patients with established cardiovascular pathology.

Author	Year	Country	Sample Size	Age	Diagnostic standard	Type of CVD	AHI cut-off	Prevalence		OR (95% CI)	Adjusted for
Vazir A. et al.	2006	UK	55	61 ± 12	PSG	CHF	5	80%		–	–
							15	53%			
							30	22%			
Otero L. et al.	2016	Colombia	834	40–80	PSG	CAD. AF	5	overall 91%		OR: 5.52 (2.9–10.7) for OSA	Not adjusted
										OR: 2.44 (1.2–5.2) for CSA	
Strotmann J. et al.	2018	Germany	211	68.7 ± 8.6	PSM	AF	5	93.4%		–	–
							15	59.7%			
Losurdo A. et al.	2018	Italy	140	66.9 ± 11.9	PSM	Ischemic stroke	10	51.40%		–	–
Zhao L.P. et al.	2014	Singapore	162	58.6 ± 0.8	PSM	CAD	15	37.9%	35.0% men	–	–
									40.3% women		
Logan A.G. et al.	2001	Canada	41	57.2 ± 1.6	PSG	RHTN	10	82.9%	95.8% men	–	–
									64.7% women		
Gessner V. et al.	2017	Germany	223	63.2 ± 11.2	PSM	Acute MI	5	85.6%	40.8% OSA	–	–
									7% CSA		
									3.1% mixed		
Prinz C. et al.	2011	Germany	63	59.5 ± 13.0	PSM	Hypertrophic Cardiomyopathy	5	82.5%	61.9% OSA	–	–
									20.6% CSA		
Lee C.H. et al.	2009	Singapore	105	53 ± 10	PSM	Acute MI	15	65.70%		–	–
Bazan V. et al.	2013	Spain	56	66 ± 11	PSG	AF	5	82%		–	–
							30	45%			
Glantz H. et al.	2013	Sweden	662	64.1 ± 8.7	PSM	CAD	15	63.7%		–	–
							30	24.6%			
Strotmann J. et al.	2017	Germany	211	68.7 ± 8.5	PSM	AF	15	57.9%	55.9% OSA	–	–
									36.5% CSA		
Muxfeldt E. et al.	2014	Brazil	422	62.4 ± 9.9	PSG	RHTN	5	82.2%		–	–
							15	55.5%			
Paulino A. et al.	2008	France	316	59 ± 3	PSM	CHD	10	81%	56% OSA	–	–
									25% CSA		
NorAdina A.T. et al.	2006	Malaysia	28	60.3 ± 8.9	PSM	Ischemic stroke	5	92.8%		–	–
							10	78.5%			
							15	44.8%			
							20	37.7%			
Redeker N.S. et al.	2010	USA	170	60.3 ± 16.8	PSG	CHD	5	84.1%		–	–
Albuquerque F.N. et al.	2011	USA	151	69.1 ± 11.7	PSG	AF	5	78.1%		–	–
							15	52.3%			
							30	29.1%			
Brooks D. et al.	2010	USA	45	67 ± 12	PSG	Stroke	10	91%		–	–
Lutohin G.M.	2016	Russia	54	66 (57; 72)	PSM	Ischemic stroke	5	92%	81.5% OSA	–	–
									11.1% CSA		
Abumuamar A.M. et al.	2018	Canada	100	63.6 ± 13.3	PSG	AF	5	85%		–	–
Boulos M.I. et al.	2016	Canada	102	68.7 ± 13.7	PSM	Stroke/TIA	5	63.40%		–	–
Hoyer F.F. et al.	2010	Germany	46	65 ± 7	PSM	AF	5	67%		–	–
Cai A. et al.	2018	China	1157	56.6 ± 11.7	PSG	RHTN	5	33.1%		OR: 1.049 (1.021–1.079)	Age, male sex, neck girth, BMI, mean SaO2 level, serum uric acid level, presence of diabetes mellitus and CHD.
Koo B. B. et al.	2016	USA	164	62 ± 11.3	PSG	Ischemic stroke	5	80.20%		men OR: 1.04 (1.00–1.09)	Age, diabetes, AF, and PHQ-8 score.
										women OR: 0.88 (0.78–0.99)	
Shah N. et al.	2013	USA	136	Median 57.2	PSM	Acute MI	5	77%		–	Age, sex, race, smoking, hyperlipidemia, hypertension, CVD history, diabetes mellitus, and baseline creatinine.
							30	10%		OR: 0.038 (0.002–0.610)	
Pedrosa R. et al.	2010	Brazil	80	47	PSM	AF	15	40%		OR: 1.07 (1.01–1.13)	Multivariate analysis
							30	21%			
Geovanini G. et al.	2016	Brazil	80	62 ± 10	PSG	Refractory angina	5	75%		–	Not adjusted
							51	25%		OR: 4.00 (1.17–13.73)	
Kohno T. et al.	2018	Japan	197	60 ± 9	PSM	AF	10	68.5%	60.9%-OSA	Hyp OR: 2.6 (1.3–5.1)	Not adjusted
									7.6%-CSA		
Sin D.D. et al.	2002	Canada	301	CSA 67.2 ± 0.9. OSA 59.4 ± 1.1	PSG	HF	10	40%		OR: 2.89 (1.25–6.73)	BMI, age, sex, mean and minimum SaO2, and LVEF.
Grimm W. et al.	2014	Germany	267	60 ± 14	PSG	Systolic HF	15	43%		–	Age, male sex, arterial hypertension, chronic kidney disease, brain natriuretic peptide, left atrial diameter, NYHA heart failure class, the use of digitalis, the lack of angiotensin-converting enzyme, inhibitors or angiotensin II receptor blockers
							30	25%		AF OR: 5.21 (1.67–16.27)	
Kumar R. et al.	2017	India	50	54.6 ± 12.49	PSG	Stroke	5	78%		OR: 1.14 (1.03–1.25)	Age, sex, BMI, and stroke severity.
							15	46%			
							30	18%			
Macdonald M. et al.	2007	USA	108	57 ± 11	PSM	CHF	15	61%	30% OSA	AF: OR: 11.56 (1.43–93.02) worse functional class of HF: OR: 2.77 (1.14–6.73)	Male sex, age >60 years, BMI, and LVEF.
									31% CSA		
Cadilhac D. A. et al.	2005	Australia	78	63.5 ± 14.7	PSG	Stroke	5	81%		–	Age, neck circumference and stroke severity.
							15	64.4%		OR: 4.15 (1.05–16.38)	
Braga B. et al.	2007	Brazil	84	60.5 ± 9.5	PSG	AF	10	81.60%		OR: 2.87 (1.07–7.70)	Not adjusted
Bekfani T. et al.	2020	Germany	111	67.6 ± 10.2	PSG	HF	5	66.7% (OSA 42.3%,CSA 21.6%, Mixed 2.7%)			

*AF* atrial fibrillation, *AHI* apnea-hypopnea index, *BMI* body mass index, *CAD* coronary artery disease, *CAI* central apnea index, *CHD* coronary heart disease, *CHF* congestive heart failure, *CSA* central sleep apnea, *CVD* cardiovascular disease, *HF* heart failure, *HR* hazard ratio, *LVEF* left ventricle ejection fraction, *MACCEs* major adverse cardiac and cerebrovascular events, *MI* myocardial infarction, *OSA* obstructive sleep apnea, *OR* odds ratio, *PHQ-8* eight-item Patient Health Questionnaire depression scale, *PSM* portable sleep monitor, *PSG* polysomnography, *RHTN* resistant hypertension, *TIA* transient ischemic attack.

Cross-sectional studies: characteristics, outcomes and results.

**Table 5:** Association of sleep-disordered breathing and cardiovascular disease in patients with established SDB.

Author	Year	Country	Study Design	Sample Size	Diagnostic Standard	Outcome	Results
Gunbatar H. et al.	2016	Turkey	cross-sectional	56	PSG	Silent prestroke damage	OR: 3.7 (1.2–11.9)
Davies C.WH et al.	2000	UK	case-control	90	PSM	Arterial hypertension	High SBP OR: 9.2 (2.3–16.1) High DBP OR: 7.2 (3.7−10.6)
Chang Chih-Cheng et al.	2014	Taiwan	case-control	149805	PSG	New diagnosis of stroke, and death.	HR: 1.19 (1.09–1.30)
Mansukhani M.P. et al.	2013	USA	case-control	108	PSG	Ischemic stroke	OR: 5.34 (1.79–17.29)
Won C.H. et al.	2012	USA	cohort	281	PSG	All-cause mortality	HR: 1.72 (1.01–2.91)

Study characteristics, outcomes and results.

*DBP* diastolic blood pressure, *HR* hazard ratio, *OR* odds ratio, *PSM* portable sleep monitor, *PSG* polysomnography, *SBP* systolic blood pressure.

SDB was assessed by overnight polysomnography in 51 studies and by validated portable diagnostic devices in 28 others. All studies screened their study populations for SDB prior to the outcomes being obtained.

The definition of SDB was based on a standardized assessment of AHI in all studies except one, where RDI was used^7^. The AHI cut-off value varied across studies from 5 to 51. In most studies, AHI ≥ 15 was used as the cut-off value for moderate-to-severe SDB.

The median follow-up duration for cohort studies and case-controls was from 6 months to 24 years. The number of patients varied between 28 and 30719, with a mean age range from 47 ± 8 to 76.3 ± 5.5 years. We did not consider whether SDB was treated during the study period. Most of the studies reported adjusted risk estimates for the primary endpoint. The potential confounding factors were age, sex, body mass index, hypercholesterolemia, hypertension, diabetes, smoking and others. In studies when authors reported both the unadjusted and adjusted risk, we used only the adjusted risk for the pooled analysis.

The types of cardiovascular pathology studied were atrial fibrillation, coronary artery disease, coronary heart disease, heart failure, myocardial infarction, resistant hypertension, transient ischemic attack (TIA) and stroke. Longitudinal studies assessed adverse cardiovascular outcomes and all-cause mortality.

### Quality assessment

Supplementary Table 1 reports the evaluation of each item from the Quality Assessment Tool for Studies (see Supplementary Information file). The quality of the reports was heterogeneous, with scores between 4–9 for cross-sectional studies, 8–12 for cohort studies and 7–10 for case-controls. In many reports, several items were missed, such as a sample size justification and power description, a definition of the potential risk of bias and a description if the outcome assessors were blinded to the exposure status of the participants.

### Association of SDB and CVD in an unselected population

**Fig. 2:**
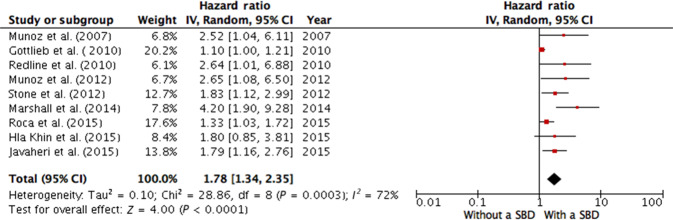
Meta-analysis of association between sleep-disordered breathing and risk of cardiovascular disease (hazard ratios) in an unselected general population. Munoz et al. (2007) AHI cut-off 30, outcome—ischemic stroke; Gottlieb et al. (2010) AHI cut-off 15, outcome—coronary heart disease; Redline et al. (2010) AHI cut-off 15, outcome—incident stroke; Munoz et al. (2012) CAI cut-off 1, outcome—ischemic stroke; Stone et al. (2012) AHI cut-off 5, outcome—incident stroke; Marshall et al. (2014) AHI cut-off 15, outcome—all-cause mortality; Roca et al. (2015): AHI cut-off 15, outcome—all-cause mortality; Hla Khin et al. (2015) AHI cut-off 15, outcome—coronary heart disease and heart failure; Javaheri et al. (2015) CAI cut-off 5, outcome—incident heart failure. AHI apnea-hypopnea index, CAI central apnea index, CI confidence interval, IV inverse variance, SDB sleep-disordered breathing.

**Fig. 3:**
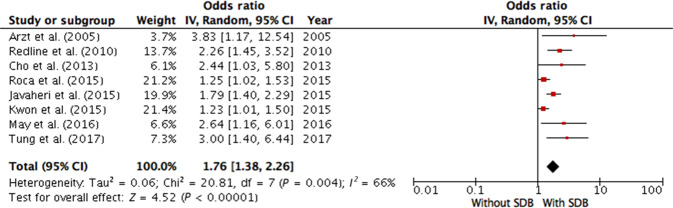
Meta-analysis of association between sleep-disordered breathing and risk of cardiovascular disease (odds ratios) in an unselected general population. Arzt et al. (2005) AHI cut-off 20, outcome—incident stroke; Redline et al. (2010) AHI cut-off 15, outcome—incident stroke; Cho et al. (2013) AHI cut-off 15, outcome—cerebral infarction; Roca et al. (2015): AHI cut-off 15, outcome—all-cause mortality; Javaheri et al. (2015) CAI cut-off 5, outcome—incident heart failure; Kwon et al. (2015) AHI cut-off 15, outcome—incident atrial fibrillation; May et al. (2016) AHI cut-off 15, outcome—incident atrial fibrillation; Tung et al. (2017) CAI cut-off 5, outcome—incident atrial fibrillation. AHI apnea-hypopnea index, CAI central apnea index, CI confidence interval, IV inversed variance, SDB sleep-disordered breathing.

Fourteen studies with 27718 participants (the mean age ranged from 47 to 76.3 years) were included in the meta-analysis to explore the associations of SDB with CVD in a general unselected population^4,5,7,13–16,25–31^ (Table 1). Due to the clinical and statistical heterogeneity of the studies, their reported risk ratios could not be pooled into a meta-analysis into investigate the power of association. Nine studies provided adequate information on the HR and eight on the OR; they were included in two separate pooled analysis. Analyzing relative risks, we found that SDB was significantly associated with the risk of cardiovascular pathology in the general unselected population (overall HR 1.78; 95% CI 1.34–2.35), and the values of the HR varied from 1.10 to 4.20. Evidence of moderate between-study heterogeneity was found (I^2^ = 72%, *p* < 0.000, Fig. 2). After pooling the ORs, we found a similar association (overall OR 1.76; 95% CI 1.38–2.26, I^2^ = 66%, *p* < 0.000, Fig. 3) with a minimum OR of 1.23 and maximum OR of 3.83.

### Association of SDB and CVD in a population at risk of sleep apnea

**Fig. 4:**
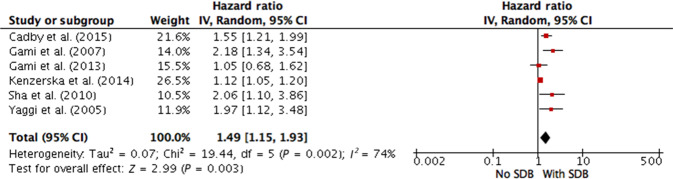
Meta-analysis of association between sleep-disordered breathing and risk of cardiovascular disease (hazard ratios) in a population at high risk of SDB. Cadby et al. (2015) AHI cut-off 5, outcome—atrial fibrillation; Gami et al. (2007) AHI cut-off 5, outcome—incident atrial fibrillation; Gami et al. (2013) AHI cut-off 20, outcome—sudden cardiac death; Kenzerska et al. (2014) AHI cut-off 5, outcome—myocardial infarction, stroke, congestive heart failure, revascularization procedure, all-cause death; Sha et al. (2010) AHI cut-off 5, outcome—cardiovascular disease; Yaggi et al. (2005) AHI cut-off 5, outcome—incident stroke/TIA or all-cause mortality. AHI apnea-hypopnea index, CI confidence interval, IV inversed variance, SDB sleep-disordered breathing.

**Fig. 5:**
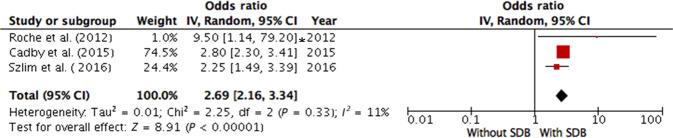
Meta-analysis of association between sleep-disordered breathing and risk of cardiovascular disease (odds ratios) in a population at high risk of SDB. Roche et al. (2002) AHI cut-off 10, outcome—nocturnal paroxysmal asystole*;* Cadby et al. (2015) AHI cut-off 5, outcome—atrial fibrillation; Szlim et al. (2016) AHI cut-off 15, outcome—nocturnal cardiac arrhythmias. AHI apnea-hypopnea index, CI confidence interval, IV inversed variance, SDB sleep-disordered breathing. *-calculated manually.

Eight studies^32–39^ were performed in patients with a high probability of having SDB. Six of the studies reported the HR, and three of the studies reported the OR; they were included in a separate pooled analysis (Table 2). SDB was also found to be associated with comorbid cardiovascular pathology in the random effects analysis (overall HR 1.49; 95% CI 1.15–1.93; overall OR 2.62; 95% CI 2.09–3.16). There was moderate evidence of between-study heterogeneity for the HR (I^2^ = 74%, *p* = 0.003, Fig. 4) and no evidence of heterogeneity for the OR (I^2^ = 11%, *p* < 0.000, Fig. 5).

### The prevalence and risk of SDB in patients with an established CVD diagnosis

We included fifty one studies^8–10,12,40–85^, in patients with various cardiovascular pathology in our review (Tables 3 and 4). The prevalence of SDB reported in these studies varied widely. Overall, SDB measured as AHI > 5 events/h ranged from 19%^43^ to 93.4%^54^. At the clinically important AHI level over 15 events/h, the prevalence of SDB in cardiovascular patients varied from 37.9%^56^ to 65.7%^47^. The highest prevalence was found in patients with myocardial infarction^47^, coronary artery disease^61^ and atrial fibrillation^54^. The incidence of major adverse events was significantly higher in cardiovascular patients with comorbid SDB than in those with only CVD (HR ranged from 1.02^9^ to 11.08^44^. Due to the clinical heterogeneity of the studies of various cardiovascular diseases, their reported prevalence and power of association could not be compared.

### The risk of CVD in patients with an established SDB diagnosis

We found five studies^11,86–89^ in patients with SDB (Table 5). In patients with established SDB diagnoses according to the validated instrumental measurement, there was a high risk for the incident cardiovascular events of different kinds. The results could not be pooled due to the important clinical heterogeneity.

### Publication bias

It was difficult to assess for publication bias due to the low number of studies in each subgroup. We tested for evidence of publication bias based on visual inspection of the funnel plot (Supplementary Figs. 1–3). There was no evidence of publication bias for the association of SDB and CVD in the general population (HR and OR). However, the funnel plot was asymmetrical for the association of SDB and CVD in the population at risk. We could therefore not rule out publication bias.

## Discussion

Our main finding was that SDB was strongly associated with CVD. In the general population as well as in patients with an already diagnosed cardiovascular pathology, the presence of SDB significantly increases the risk of adverse cardiovascular outcomes and all-cause mortality. Considering the higher prevalence of SDB in cardiovascular patients (up to 93–96%^54,57^) and the higher risk of adverse cardiovascular events, it may be reasonable to screen for possible SDB in cardiovascular patients.

Our results suggest that the presence of SDB increases the risk of cardiovascular events 1.5 times in the general population and 2.6 times in the population at risk. The prevalence of OSA^90^ in the general population, as well as the correlation between OSA and risk of certain cardiovascular events, has already been established in earlier reviews^20–22^. In the general population, OSA was significantly associated with stroke and IHD. However, these literature reviews presented data only on one type of SDB and studied a particular type of cardiovascular pathology as an outcome and/or the risk in patients with preexisting cardiovascular pathology. In contrast, the present meta-analysis focused on all types of cardiovascular pathology and SDB. Regarding adverse cardiovascular outcomes and mortality, our results were consistent with those reported in the general population^20,91^. However, due to the methodological and clinical heterogeneity of the studies, the results could not be pooled in most of the meta-analyses.

In our review, we found that in patients with established cardiovascular pathology, SDB significantly increased the risk of new adverse cardiovascular events (up to 3.58 times). The results are consistent with other recent literature reviews and meta-analyses, with four-fold higher odds for AF^92^ and a threefold higher risk for ischemic stroke^93^. We did not find a high-quality meta-analysis on other types of cardiovascular pathology.

The pathophysiological mechanisms of SDB and associated cardiovascular risk are complex and vary between patients. Obstructive and central apnea may induce severe intermittent hypoxemia and CO_2_ retention during sleep, with oxygen saturation sometimes dropping to 60%, disrupting the normal structured autonomic and hemodynamic responses to sleep^94^. Apnea occurs repetitively through the night and is accompanied by increases in sympathetic activity to peripheral blood vessels and consequent vasoconstriction^95^. Nocturnal apnea initiates a range of pathophysiological mechanisms that may act to promote cardiac and vascular disease. These mechanisms include sympathetic activation, oxidative stress, cardiovascular variability, increased release of vasoactive and trophic substances, inflammation, endothelial dysfunction and thrombosis^96^. Thus, sleep apnea may initiate, promote, or exacerbate hypertension, coronary artery disease, arrhythmias, stroke, and heart failure^97^. Compared with the general population, the prevalence of SDB is higher in patients with cardiovascular conditions, such as hypertension (33–83%^57,73^), heart failure (40–61%^78,81^), atrial fibrillation (67–93.4%^54,72^), and stroke (63–92%^65,71^). However, the coexistence of these conditions with SDB does not prove causality, and potential confounding variables may intervene.

In the European Society of Cardiology Guidelines for the management of atrial fibrillation^98^, it is recommended that all AF patients be interrogated for symptoms and clinical signs of OSA. In addition, the treatment of an associated OSA should be optimized to reduce AF recurrences and improve AF treatment results (class of recommendation IIa). In addition, the updated Guideline for the Management of Heart Failure (American College of Cardiology/American Heart Association)^99^ stated that in patients with NYHA class II–IV HF and suspicion of SDB or excessive daytime sleepiness, a formal sleep assessment is reasonable (class of recommendation IIa). Thus, it is necessary to screen for possible SDB in patients with severe associated cardiovascular pathology, although the evidence of treatment benefits is still scarce.

This is the first comprehensive systematic review of the literature on the association between SDB and all kinds of cardiovascular pathology in adults in all regions of the world. Previous systematic reviews were limited to certain types of CVD or SDB or to selected populations.

The most significant strength of our systematic review is that we included both types of studies: those that measured the association in an unselected general population (or age-/sex-specific subgroups) and in a population at risk of SDB; and those that focused on a select population (with suspected sleep disorder or with diagnosed CVD). We included studies in which sleep apnea was measured using standard objective procedures.

Due to heterogeneity across studies and outcomes, there was a limited scope for conducting a meta-analysis. We have generally relied on adjusted data, but in some reports, risk indices were not adjusted for traditional risk factors or were adjusted for only some risk factors. We realize that confounding and differences in patient selection comprise a possible source of bias. Another limitation is that the range of AHI cut-off values varies across studies. Unfortunately, it was not possible to conduct separate subgroup meta-analyses for the associations between SDB severity (i.e., mild, moderate, and severe, according to AHI) and cardiovascular outcomes because of small sample sizes. When data concerning SDB severity were reported, we included in the pooled analysis the HR and OR related to the most severe forms of SDB (i.e., those with the highest AHI cut-off value).

In conclusion, we found an association between sleep-disordered breathing and cardiovascular disease. Notably, in patients with existing SDB pathology, the risk of new adverse cardiovascular events is high. However, the relationship between CVD and SDB is likely to be bidirectional. Thus, more large-scale studies are needed to better understand this association and to decide whether screening for possible SDB in cardiovascular patients is indicated and clinically relevant.

## Supplementary information

Supplementary Information

Reporting Summary

## Data Availability

The search concepts are available in the supplement.

## References

[1] Young T (1993). The occurrence of sleep-disordered breathing among middle-aged adults. N. Engl. J. Med..

[2] Berry RB (2015). AASM scoring manual version 2.2 updates: new chapters for scoring infant sleep staging and home sleep Apnea testing. J. Clin. Sleep. Med..

[3] Young T (2008). Sleep disordered breathing and mortality: eighteen-year follow-up of the Wisconsin sleep cohort. Sleep.

[4] Arzt M, Young T, Finn L, Skatrud JB, Bradley TD (2005). Association of sleep-disordered breathing and the occurrence of stroke. Am. J. Respiratory Crit. Care Med..

[5] Tung P (2017). Obstructive and central sleep Apnea and the risk of incident atrial fibrillation in a community cohort of men and women. J. Am. Heart Association.

[6] Javaheri S (2016). Association between obstructive sleep apnea and left ventricular structure by age and gender: The multi-ethnic study of atherosclerosis. Sleep.

[7] Marshall NS, Wong KKH, Cullen SRJ, Knuiman MW, Grunstein RR (2014). Sleep apnea and 20-year follow-up for all-cause mortality, stroke, and cancer incidence and mortality in the Busselton Health Study cohort. J. Clin. Sleep. Med.: JCSM: Off. Publ. Am. Acad. Sleep. Med..

[8] Khayat R (2015). Sleep disordered breathing and post-discharge mortality in patients with acute heart failure. Eur. Heart J..

[9] Emdin M (2017). Prognostic significance of central Apneas throughout a 24-hour period in patients with heart failure. J. Am. Coll. Cardiol..

[10] Shah N (2013). Obstructive sleep apnea and acute myocardial infarction severity: ischemic preconditioning?. Sleep. Breath. = Schlaf Atm..

[11] Chang C-C (2014). High incidence of stroke in young women with sleep apnea syndrome. Sleep. Med..

[12] Parra O (2004). Sleep-related breathing disorders: impact on mortality of cerebrovascular disease. Eur. Respiratory J..

[13] Hla KM (2015). Coronary heart disease incidence in sleep disordered breathing: the Wisconsin Sleep Cohort Study. Sleep.

[14] Gottlieb, D. et al. Prospective study of obstructive sleep Apnea and incident coronary heart disease and heart failure: the sleep heart health study. *Circulation* **122**, 352–360 (2010). 10.1161/CIRCULATIONAHA.109.901801 PMC3117288 20625114

[15] Stone KL (2016). Sleep disordered breathing and risk of stroke in older community-dwelling men. Sleep.

[16] Roca, G. Q. et al. Sex-specific association of sleep apnea severity with subclinical myocardial injury, ventricular hypertrophy, and heart failure risk in a community-dwelling cohort. *Circulation* **132**, 1329–1337 (2015). 10.1161/CIRCULATIONAHA.115.016985 PMC4596785 26316620

[17] Punjabi NM, Newman AB, Young TB, Resnick HE, Sanders MH (2008). Sleep-disordered breathing and cardiovascular disease: an outcome-based definition of hypopneas. Am. J. Respir. Crit. Care Med..

[18] Somers VK (2008). Sleep apnea and cardiovascular disease: an American Heart Association/american College Of Cardiology Foundation Scientific Statement from the American Heart Association Council for High Blood Pressure Research Professional Education Committee, Council on Clinical Cardiology, Stroke Council, and Council On Cardiovascular Nursing. In collaboration with the National Heart, Lung, and Blood Institute National Center on Sleep Disorders Research (National Institutes of Health). Circulation.

[19] Duran-Cantolla J (2010). Continuous positive airway pressure as treatment for systemic hypertension in people with obstructive sleep apnoea: randomised controlled trial. BMJ (Clin. Res. ed.).

[20] Loke YK, Brown JW, Kwok CS, Niruban A, Myint PK (2012). Association of obstructive sleep apnea with risk of serious cardiovascular events: a systematic review and meta-analysis. Circulation Cardiovascular Qual. Outcomes.

[21] Porto F, Sakamoto YS, Salles C (2017). Association between obstructive sleep Apnea and myocardial infarction: a systematic review. Arquivos Brasileiros de. Cardiologia..

[22] Xie W, Zheng F, Song X (2014). Obstructive sleep apnea and serious adverse outcomes in patients with cardiovascular or cerebrovascular disease: a PRISMA-compliant systematic review and meta-analysis. Medicine.

[23] Moher D, Liberati A, Tetzlaff J, Altman DG (2009). Preferred reporting items for systematic reviews and meta-analyses: the PRISMA statement. PLoS Med..

[24] Stroup DF (2000). Meta-analysis of observational studies in epide.miology: a proposal for reporting. Meta-analysis Of Observational Studies in Epidemiology (MOOSE) group. Jama.

[25] Javaheri, S. et al. Sleep-disordered breathing and incident heart failure in older men. *Am J Respir. Crit. Care Med.* **193**, 561–568 (2016). 10.1164/rccm.201503-0536OC PMC4824922 26502092

[26] Redline S (2010). Obstructive sleep apnea-hypopnea and incident stroke: the sleep heart health study. Am. J. Respir. Crit. Care Med..

[27] Munoz R (2006). Severe sleep apnea and risk of ischemic stroke in the elderly. Stroke.

[28] Munoz R (2012). Central sleep apnea is associated with increased risk of ischemic stroke in the elderly. Acta Neurologica Scandinavica..

[29] May AM (2016). Central sleep-disordered breathing predicts incident atrial fibrillation in older men. Am. J. Respiratory Crit. Care Med..

[30] Cho ER (2013). Obstructive sleep apnea as a risk factor for silent cerebral infarction. J. Sleep. Res..

[31] Kwon, Y. et al. Association of sleep characteristics with atrial fibrillation: the Multi-Ethnic Study of Atherosclerosis. *Thorax* **70**, 873–879 (2015). 10.1136/thoraxjnl-2014-206655 PMC5495463 25986436

[32] Cadby, G. et al. Severity of OSA is an independent predictor of incident atrial fibrillation hospitalization in a large sleep-clinic cohort. *Chest* **148**, 945–952 (2015). 10.1378/chest.15-0229 25927872

[33] Gami AS (2013). Obstructive sleep apnea and the risk of sudden cardiac death: a longitudinal study of 10,701 adults. J. Am. Coll. Cardiol..

[34] Shah NA, Yaggi HK, Concato J, Mohsenin V (2010). Obstructive sleep apnea as a risk factor for coronary events or cardiovascular death. Sleep. Breath. = Schlaf Atm..

[35] Yaggi HK (2005). Obstructive sleep apnea as a risk factor for stroke and death. N. Engl. J. Med..

[36] Gami AS (2007). Obstructive sleep apnea, obesity, and the risk of incident atrial fibrillation. J. Am. Coll. Cardiol..

[37] Kendzerska T, Gershon AS, Hawker G, Leung RS, Tomlinson G (2014). Obstructive sleep apnea and risk of cardiovascular events and all-cause mortality: a decade-long Historical Cohort Study. PLoS Med..

[38] Selim BJ (2016). The association between nocturnal cardiac arrhythmias and sleep-disordered breathing: The DREAM study. J. Clin. Sleep. Med..

[39] Roche F (2003). Relationship among the severity of sleep apnea syndrome, cardiac arrhythmias, and autonomic imbalance. Pacing Clin. Electrophysiology: Pace..

[40] Sano K (2013). Central sleep apnoea and inflammation are independently associated with arrhythmia in patients with heart failure. Eur. J. Heart Fail..

[41] Mooe T, Franklin KA, Holmstrom K, Rabben T, Wiklund U (2001). Sleep-disordered breathing and coronary artery disease: long-term prognosis. Am. J. Respiratory Crit. Care Med..

[42] Leao S (2016). Effect of obstructive sleep apnea in acute coronary syndrome. Am. J. Cardiol..

[43] Shah RV (2014). Obesity and sleep apnea are independently associated with adverse left ventricular remodeling and clinical outcome in patients with atrial fibrillation and preserved ventricular function. Am. Heart J..

[44] Ponsaing LB, Iversen HK, Jennum P (2017). Polysomnographic indicators of mortality in stroke patients. Sleep. Breath..

[45] Lee C-H (2011). Severe obstructive sleep apnea and outcomes following myocardial infarction. J. Clin. Sleep. Med.: JCSM: Off. Publ. Am. Acad. Sleep. Med..

[46] Javaheri S, Caref EB, Chen E, Tong KB, Abraham WT (2011). Sleep apnea testing and outcomes in a large cohort of Medicare beneficiaries with newly diagnosed heart failure. Am. J. Respir. Crit. Care Med..

[47] Lee C-H (2009). Obstructive sleep apnea in patients admitted for acute myocardial infarction. Prevalence, predictors, and effect on microvascular perfusion. Chest.

[48] Bolotova MN (2008). Obstructive sleep apnea and a cardiovascular risk in patients with arterial hypertension. Vestn. Rentgenologii i Radiologii..

[49] Uchoa C (2015). Impact of OSA on cardiovascular events after coronary artery bypass surgery. Chest.

[50] Szymanski FM (2015). Presence and severity of obstructive sleep apnea and remote outcomes of atrial fibrillation ablations - a long-term prospective, cross-sectional cohort study. Sleep. Breath. = Schlaf Atm..

[51] Zhao L-P (2015). Relationship between severity of obstructive sleep apnea and adverse cardiac outcomes in non-diabetic patients presenting with myocardial infarction. Eur. Arch. Oto-Rhino-Laryngol.: Off. J. Eur. Federation Oto-Rhino-Laryngological Societies (EUFOS): affiliated Ger. Soc. Oto-Rhino-Laryngol. - Head. Neck Surg..

[52] Vazir A (2007). A high prevalence of sleep disordered breathing in men with mild symptomatic chronic heart failure due to left ventricular systolic dysfunction. Eur. J. Heart Fail..

[53] Otero L, Hidalgo P, González R, Morillo CA (2016). Association of cardiovascular disease and sleep apnea at different altitudes. High. Alt. Med. Biol..

[54] Strotmann J (2018). Characteristics of sleep-disordered breathing in patients with atrial fibrillation and preserved left ventricular ejection fraction. Clin. Res. Cardiology: Off. J. Ger. Card. Soc..

[55] Losurdo A (2018). Dysphagia and obstructive sleep apnea in acute, first-ever, Ischemic stroke. J. Stroke Cerebrovasc. Dis.: Off. J. Natl. Stroke Assoc..

[56] Zhao L-P (2014). Effects of gender on the prevalence of obstructive sleep apnea in patients with coronary artery disease. J. Clin. Sleep. Med.: JCSM: Off. Publ. Am. Acad. Sleep. Med..

[57] Logan A (2001). High prevalence of unrecognized sleep apnoea in drug-resistant hypertension. J. Hypertens..

[58] Gessner V, Bitter T, Horstkotte D, Oldenburg O, Fox H (2017). Impact of sleep-disordered breathing in patients with acute myocardial infarction: a retrospective analysis. J. Sleep. Res..

[59] Prinz C (2011). Incidence of sleep-disordered breathing in patients with hypertrophic cardiomyopathy. Congest Heart Fail..

[60] Bazan V (2013). Obstructive sleep apnea in patients with typical atrial flutter: prevalence and impact on Arrhythmia control outcome. Chest.

[61] Glantz H (2013). Occurrence and predictors of obstructive sleep apnea in a revascularized coronary artery disease cohort. Ann. Am. Thorac. Soc..

[62] Strotmann J (2017). Predominant obstructive or central sleep apnea in patients with atrial fibrillation: influence of characterizing apneas versus apneas and hypopneas. Sleep. Med..

[63] Muxfeldt E, Margallo V, Guimaraes G, Salles G (2014). Prevalence and associated factors of obstructive sleep apnea in patients with resistant hypertension. Am. J. Hypertens..

[64] Paulino A (2009). Prevalence of sleep-disordered breathing in a 316-patient French cohort of stable congestive heart failure. Arch. Cardiovascular Dis..

[65] Noradina AT, Hamidon BB, Roslan H, Raymond AA (2006). Risk factors for developing sleep-disordered breathing in patients with recent ischaemic stroke. Singap. Med. J..

[66] Redeker NS (2010). Sleep disordered breathing, daytime symptoms, and functional performance in stable heart failure. Sleep.

[67] Albuquerque F (2012). Sleep-disordered breathing and excessive daytime sleepiness in patients with atrial fibrillation. Chest.

[68] Brooks D (2010). Sleep-disordered breathing in patients enrolled in an inpatient stroke rehabilitation program. Arch. Phys. Med. Rehabilitation..

[69] Lutokhin, G. M., Geraskina, L. A. & Fonyakin, A. V. [Sleep-disordered breathing syndrome in acute ischemic stroke]. Синдром нарушения дыхания во сне при ишемическом инсульте. *Zhurnal nevrologii i psikhiatrii imeni SS Korsakova* **116**, 14–20 (2016). 10.17116/jnevro201611612214-20 28300797

[70] Abumuamar AM, Dorian P, Newman D, Shapiro CM (2018). The prevalence of obstructive sleep apnea in patients with atrial fibrillation. Clin. Cardiol..

[71] Boulos MI (2017). Unattended Hospital and home sleep apnea testing following cerebrovascular events. J. Stroke Cerebrovasc. Dis.: Off. J. Natl. Stroke Assoc..

[72] Hoyer FF (2010). High prevalence of obstructive sleep apnea in patients with resistant paroxysmal atrial fibrillation after pulmonary vein isolation. J. Interventional Card. Electrophysiology: Int. J. Arrhythm. Pacing.

[73] Cai A (2018). Joint effects of obstructive sleep apnea and resistant hypertension on chronic heart failure: a cross-sectional study. Int. J. Cardiol..

[74] Koo BB (2016). Observational study of obstructive sleep apnea in wake-up stroke: The SLEEP TIGHT study. Cerebrovasc. Dis..

[75] Pedrosa R (2010). Obstructive sleep apnea is common and independently associated with atrial fibrillation in patients with hypertrophic cardiomyopathy. Chest.

[76] Geovanini G (2016). Obstructive sleep apnoea is associated with myocardial injury in patients with refractory angina. Heart.

[77] Kohno T (2018). Prevalence and clinical characteristics of obstructive- and central-dominant sleep apnea in candidates of catheter ablation for atrial fibrillation in Japan. Int J. Cardiol..

[78] Sin DD (2003). Relationship of systolic BP to obstructive sleep apnea in patients with heart failure. Chest.

[79] Grimm W (2015). Severe central sleep apnea is associated with atrial fibrillation in patients with left ventricular systolic dysfunction. Pacing Clin. Electrophysiology: Pace..

[80] Kumar R, Suri JC, Manocha R (2017). Study of association of severity of sleep disordered breathing and functional outcome in stroke patients. Sleep. Med..

[81] MacDonald M, Fang J, Pittman SD, White DP, Malhotra A (2008). The current prevalence of sleep disordered breathing in congestive heart failure patients treated with beta-blockers. J. Clin. Sleep. Med.: JCSM: Off. Publ. Am. Acad. Sleep. Med..

[82] Cadilhac DA (2005). Sleep disordered breathing in chronic stroke survivors. A study of the long term follow-up of the SCOPES cohort using home based polysomnography. J. Clin. Neurosci..

[83] Braga B (2009). Sleep-disordered breathing and chronic atrial fibrillation. Sleep. Med..

[84] Bekfani T (2020). Heart failure and sleep-disordered breathing: susceptibility to reduced muscle strength and preclinical congestion (SICA-HF cohort). ESC Heart Fail..

[85] Fan J (2019). Association of obstructive sleep apnea with cardiovascular outcomes in patients with acute coronary syndrome. J. Am. Heart Assoc..

[86] Gunbatar H (2016). A silent pre-stroke damage: Obstructive sleep apnea syndrome. Int. J. Clin. Exp. Med..

[87] Davies C (2000). Case-control study of 24 h ambulatory blood pressure in patients with obstructive sleep apnoea and normal matched control subjects. Thorax.

[88] Mansukhani MP (2013). The association between atrial fibrillation and stroke in patients with obstructive sleep apnea: a population-based case-control study. Sleep. Med..

[89] Won CH (2013). Severe obstructive sleep apnea increases mortality in patients with ischemic heart disease and myocardial injury. Sleep. Breath. = Schlaf Atm..

[90] Senaratna CV (2017). Prevalence of obstructive sleep apnea in the general population: a systematic review. Sleep. Med. Rev..

[91] Wang X, Fan JY, Zhang Y, Nie SP, Wei YX (2018). Association of obstructive sleep apnea with cardiovascular outcomes after percutaneous coronary intervention: a systematic review and meta-analysis. Medicine.

[92] Geovanini GR, Lorenzi-Filho G (2018). Cardiac rhythm disorders in obstructive sleep apnea. J. Thorac. Dis..

[93] King S, Cuellar N (2016). Obstructive sleep apnea as an independent stroke risk factor: a review of the evidence, stroke prevention guidelines, and implications for neuroscience nursing practice. J. Neurosci. Nurs.: J. Am. Assoc. Neurosci. Nurses.

[94] Somers VK, Dyken ME, Mark AL, Abboud FM (1993). Sympathetic-nerve activity during sleep in normal subjects. N. Engl. J. Med..

[95] Somers VK, Mark AL, Zavala DC, Abboud FM (1989). Contrasting effects of hypoxia and hypercapnia on ventilation and sympathetic activity in humans. J. Appl. Physiol. (Bethesda, Md: 1985)..

[96] Somers VK (2008). Sleep apnea and cardiovascular disease: an American Heart Association/American College of Cardiology Foundation Scientific Statement from the American Heart Association Council for High Blood Pressure Research Professional Education Committee, Council on Clinical Cardiology, Stroke Council, and Council on Cardiovascular Nursing. J. Am. Coll. Cardiol..

[97] Bradley TD, Floras JS (2009). Obstructive sleep apnoea and its cardiovascular consequences. Lancet (Lond., Engl.)..

[98] Kirchhof P (2016). 2016 ESC Guidelines for the management of atrial fibrillation developed in collaboration with EACTS. Eur. Heart J..

[99] Yancy CW (2017). 2017 ACC/AHA/HFSA focused update of the 2013 ACCF/AHA guideline for the management of heart failure: a report of the American College of Cardiology/American Heart Association task force on clinical practice guidelines and the heart failure society of America. J. Card. Fail..

