# Supplementary material for: A systematic review on the association of sleep-disordered breathing with cardiovascular pathology in adults
Source: NPJ Prim Care Respir Med. 2022 Oct 17;32:41. doi: 10.1038/s41533-022-00307-6 (PMC9576790; doi:10.1038/s41533-022-00307-6)

**Supplementary table 1: The quality assessment of all articles based on Quality Assessment Tool for Case-Control, Cohort and Cross-sectional Studies**					
**TITLE**	**AUTHOR**	**YEAR**	**DESIGN**	**STUDIE POPULATION**	**QUALITY**
Severe Obstructive Sleep Apnea and Outcomes Following Myocardial Infarction	Lee Chi-Hang	2011	cohort	CVD screened for SDB	12
Central sleep apnoea and inflammation are independently associated with arrhythmia in patients with heart failure	Kan Sano	2013	cohort	CVD screened for SDB	12
Coronary heart disease incidence in sleep disordered breathing: the Wisconsin Sleep Cohort Study	Hla Khin Mae	2015	cohort	General population	12
Sleep disordered breathing and post-discharge mortality in patients with acute heart failure	Khayat R.	2014	cohort	CVD screened for SDB	11
A Prospective Study of Obstructive Sleep Apnea and Incident Coronary Heart Disease and Heart Failure: The Sleep Heart Health Study	Gottlieb D.J.	2010	cohort	General population	11
Effect of obstructive sleep apnea in acute coronary syndrome	Silvia L.	2016	cohort	CVD screened for SDB	11
Obstructive and Central Sleep Apnea and the Risk of Incident Atrial Fibrillation in a Community Cohort of Men and Women	Tung P.	2017	cohort	General population	11
Obstructive Sleep Apnea and Risk of Cardiovascular Events and All-Cause Mortality: A Decade-Long Historical Cohort Study	Kendzerska T.	2014	cohort	general population at risk of SDB	11
Obstructive sleep apnea and the risk of sudden cardiac death: A longitudinal study of 10,701 adults	Gami A.S.	2013	cohort	general population at risk of SDB	11
Obstructive sleep apnea as a risk factor for stroke and death	Yaggi H. K.	2005	cohort	general population at risk of SDB	11
Obstructive sleep apnea-hypopnea and incident stroke: the sleep heart health study	Redline S.	2010	cohort	General population	11
Polysomnographic indicators of mortality in stroke patients	Ponsaing L. B.	2017	cohort	CVD screened for SDB	11
High incidence of stroke in young women with sleep apnea syndrome	Chang Chih-Cheng	2014	case-control	SDB screened For CVD	10
Prognostic Significance of Central Apneas Throughout a 24-Hour Period in Patients With Heart Failure	Emdin M.	2017	cohort	CVD screened for SDB	10
Severe obstructive sleep apnea increases mortality in patients with ischemic heart disease and myocardial injury	Won C.H.	2012	cohort	SDB screened For CVD	10
Sleep-disordered Breathing and Coronary Artery Disease Long-term Prognosis	Mooe T.	2001	cohort	CVD screened for SDB	10
Impact of OSA on Cardiovascular Events After Coronary Artery Bypass Surgery	Uchoa C.	2015	cohort	CVD screened for SDB	10

Obesity and sleep apnea are independently associated with adverse left ventricular remodeling and clinical outcome in patients with atrial fibrillation and preserved ventricular function	Shah R.V.	2014	cohort	CVD screened for SDB	10
Obstructive sleep apnea, obesity, and the risk of incident atrial fibrillation	Gami A.S.	2007	cohort	general population at risk of SDB	10
Presence and severity of obstructive sleep apnea and remote outcomes of atrial fibrillation ablations - a long-term prospective, cross-sectional cohort study	Szymanski F.M.	2015	cohort	CVD screened for SDB	10
Severity of OSA Is an Independent Predictor of Incident Atrial Fibrillation Hospitalization in a Large Sleep-Clinic Cohort	Cadby G.	2015	cohort	general population at risk of SDB	10
Sex-Specific Association of Sleep Apnea Severity with Subclinical Myocardial Injury, Ventricular Hypertrophy, and Heart Failure Risk in a Community Dwelling Cohort: The Atherosclerosis Risk in Communities-Sleep Heart Health Study	Roca G.Q.	2015	cohort	General population	10
Sleep Disordered Breathing and Risk of Stroke in Older Community-Dwelling Men	Stone K.L.	2015	cohort	General population	10
Sleep-disordered Breathing and Incident Heart Failure in Older Men	Javaheri S.	2015	cohort	General population	10
Obstructive Sleep Apnea and Cardiovascular Events After Percutaneous Coronary Intervention	Lee Chi-Hang	2016	cohort	CVD screened for SDB	10
Sleep-disordered breathing and chronic atrial fibrillation	Braga B.	2007	case-control	CVD screened for SDB	9
The Association between Atrial Fibrillation and Stroke in Patients with Obstructive Sleep Apnea: A Population-Based Case-Control Study	Prabhdas M.	2013	case-control	general population at risk of SDB	9
Sleep Apnea Testing and Outcomes in a Large Cohort of Medicare Beneficiaries with Newly Diagnosed Heart Failure	Javaheri S.	2010	cohort	CVD screened for SDB	9
Sleep-related breathing disorders: impact on mortality of cerebrovascular disease	Parra O.	2004	cohort	CVD screened for SDB	9
[Obstructive sleep apnea and a cardiovascular risk in patients with arterial hypertension] Обструктивное апноэ сна и риск развития сердечно-сосудистых осложнений у пациентов с артериальной гипертензией	Bolotova M. N.	2008	cohort	CVD screened for SDB	9
Central sleep apnea is associated with increased risk of ischemic stroke in the elderly	Munoz R.	2011	cohort	General population	9
Central Sleep-disordered Breathing Predicts Incident Atrial Fibrillation in Older Men	May A.M.	2016	cohort	General population	9

Obstructive sleep apnea as a risk factor for coronary events or cardiovascular death	Shah N.A.	2010	cohort	general population at risk of SDB	9
Severe Sleep Apnea and Risk of Ischemic Stroke in the Elderly	Munoz R.	2006	cohort	General population	9
Sleep Apnea and 20-Year Follow-Up for All-Cause Mortality, Stroke, and Cancer Incidence and Mortality in the Busselton Health Study Cohort	Marshall N.S.	2014	cohort	General population	9
The Association between Nocturnal Cardiac Arrhythmias and Sleep-Disordered Breathing: The DREAM Study	Selim B.J.	2016	cross-sectional	general population at risk of SDB	9
Case-control study of 24 hour ambulatory blood pressure in patients with obstructive sleep apnoea and normal matched control subjects	Davies C.WH	2000	case-control	SDB screened For CVD	8
Relationship between severity of obstructive sleep apnea and adverse cardiac outcomes in non-diabetic patients presenting with myocardial infarction	Zhao Liang-Ping	2015	cohort	CVD screened for SDB	8
High prevalence of obstructive sleep apnea in patients with resistant paroxysmal atrial fibrillation after pulmonary vein isolation	Hoyer F.F.	2010	case-control	CVD screened for SDB	7
Association of sleep characteristics with atrial fibrillation: the Multi-Ethnic Study of Atherosclerosis	Kwon Younghoon	2015	cross-sectional	General population	7
Association of Sleep-disordered Breathing and the Occurrence of Stroke	Arzt M.	2005	cross-sectional	General population	7
Impact of sleep-disordered breathing in patients with acute myocardial infarction: a retrospective analysis	Gessner V.	2017	cross-sectional	CVD screened for SDB	7
Obstructive sleep apnoea is associated with myocardial injury in patients with refractory angina	Geovanini G.	2016	cross-sectional	CVD screened for SDB	7
Sleep Disordered Breathing, Daytime Symptoms, and Functional Performance in Stable Heart Failure	Redeker N.S.	2010	cross-sectional	CVD screened for SDB	7
Study of association of severity of sleep disordered breathing and functional outcome in stroke patients	Kumar R.	2017	cross-sectional	CVD screened for SDB	7
The Current Prevalence of Sleep Disordered Breathing in Congestive Heart Failure Patients Treated with Beta-Blockers	Macdonald M.	2007	cross-sectional	CVD screened for SDB	7
The prevalence of obstructive sleep apnea in patients with atrial fibrillation	Abumuamar A.M.	2018	cross-sectional	CVD screened for SDB	7
Unattended Hospital and Home Sleep Apnea Testing Following Cerebrovascular Events	Boulos M.I.	2016	cross-sectional	CVD screened for SDB	7

Sleep disordered breathing in chronic stroke survivors. A study of the long term follow-up of the SCOPES cohort using home based polysomnography	Cadilhac D. A.	2005	cross-sectional	CVD screened for SDB	7
A high prevalence of sleep disordered breathing in men with mild symptomatic chronic heart failure due to left ventricular systolic dysfunction	Vazir P.C.	2006	cross-sectional	CVD screened for SDB	6
A silent pre-stroke damage: Obstructive sleep apnea syndrome	Gunbatar H.	2016	cross-sectional	SDB screened For CVD	6
Association of Cardiovascular Disease and Sleep Apnea at Different Altitudes	Otero L.	2016	cross-sectional	CVD screened for SDB	6
Joint effects of obstructive sleep apnea and resistant hypertension on chronic heart failure: A cross-sectional study	Anping Cai	2018	cross-sectional	CVD screened for SDB	6
Obstructive sleep apnea as a risk factor for silent cerebral infarction	Cho Eo Rin	2013	cross-sectional	General population	6
Obstructive sleep apnea in patients admitted for acute myocardial infarction. Prevalence, predictors, and effect on microvascular perfusion	Lee Chi-Hang	2009	cross-sectional	CVD screened for SDB	6
Obstructive Sleep Apnea in Patients With Typical Atrial Flutter: Prevalence and Impact on Arrhythmia Control Outcome	Bazan V.	2013	cross-sectional	CVD screened for SDB	6
Occurrence and predictors of obstructive sleep apnea in a revascularized coronary artery disease cohort	Glantz H.	2013	cross-sectional	CVD screened for SDB	6
Prevalence and Associated Factors of Obstructive Sleep Apnea in Patients with Resistant Hypertension	Muxfeldt E.	2014	cross-sectional	CVD screened for SDB	6
Relationship among the severity of sleep apnea syndrome, cardiac arrhythmias, and autonomic imbalance	Roche F.	2002	cross-sectional	SDB screened For CVD	6
Risk factors for developing sleep disordered breathing in patients with recent ischaemic stroke	NorAdina A.T.	2006	cross-sectional	CVD screened for SDB	6
Severe Central Sleep Apnea Is Associated with Atrial Fibrillation in Patients with Left Ventricular Systolic Dysfunction	GRIMM W.	2014	cross-sectional	CVD scr for SDB and another CVD	6
Dysphagia and Obstructive Sleep Apnea in Acute, First-Ever, Ischemic Stroke.	Losurdo A.	2018	cross-sectional	CVD screened for SDB	5
Effects of gender on the prevalence of obstructive sleep apnea in patients with coronary artery disease	Zhao Liang-Ping	2014	cross-sectional	CVD screened for SDB	5
High prevalence of unrecognized sleep apnoea in drug-resistant hypertension	Logan A.	2001	cross-sectional	CVD screened for SDB	5
Incidence of Sleep-Disordered Breathing in Patients With Hypertrophic Cardiomyopathy	Prinz C.	2011	cross-sectional	CVD screened for SDB	5

Observational study of obstructive sleep apnea in wake-up stroke: The SLEEP TIGHT study	Koo B. B.	2016	cross-sectional	CVD screened for SDB	5
Obstructive sleep apnea and acute myocardial infarction severity: ischemic preconditioning?	Shah N.	2013	cross-sectional	CVD screened for SDB	5
Obstructive Sleep Apnea Is Common and Independently Associated With Atrial Fibrillation in Patients With Hypertrophic Cardiomyopathy	Pedrosa R.P.	2010	cross-sectional	CVD screened for SDB	5
Predominant obstructive or central sleep apnea in patients with atrial fibrillation: influence of characterizing apneas versus apneas and hypopneas	Strotmann J.	2017	cross-sectional	CVD screened for SDB	5
Relationship of Systolic BP to Obstructive Sleep Apnea in Patients With Heart Failure	Sin Don D	2002	cross-sectional	CVD screened for SDB	5
Sleep-Disordered Breathing and Excessive Daytime Sleepiness in Patients With Atrial Fibrillation	Albuquerque F.N.	2011	cross-sectional	CVD screened for SDB	5
Sleep-disordered breathing syndrome in acute ischemic stroke	Lutohin G.M.	2016	cross-sectional	CVD screened for SDB	5
Characteristics of sleep-disordered breathing in patients with atrial fibrillation and preserved left ventricular ejection fraction	Strotmann J.	2018	cross-sectional	CVD screened for SDB	4
Prevalence and clinical characteristics of obstructive- and central-dominant sleep apnea in candidates of catheter ablation for atrial fibrillation in Japan	Kohno T.	2018	cross-sectional	CVD screened for SDB	4
Prevalence of sleep-disordered breathing in a 316-patient French cohort of stable congestive heart failure	Paulino A.	2008	cross-sectional	CVD screened for SDB	4
Sleep-Disordered Breathing in Patients Enrolled in an Inpatient Stroke Rehabilitation Program	Brooks D.	2010	cross-sectional	CVD screened for SDB	4
Heart failure and sleep-disordered breathing: susceptibility to reduced muscle strength and preclinical congestion (SICA-HF cohort)	Bekfani T	2020	Cross-sectional	CVD screened for SDB	5
Outcomes in Patients With Acute Coronary Syndrome	Fan J	2019	Cross-sectional +prospective	CVD screened for SDB + follow-up for incident CVD	6
Prognostic value of sleep apnea and nocturnal hypoxemia in patients with decompensated heart failure	Huang J	2020	Cross-sectional + prospective	CVD screened vor SDB + follow up for incident CVD	6
CVD – cardiovascular disease, SDB – sleep disordered breathing.					

Supplementary figures

Funnel plot 1

The association between sleep disordered breathing and risk of cardiovascular diseases (Hazard ratios) in an unselected general population

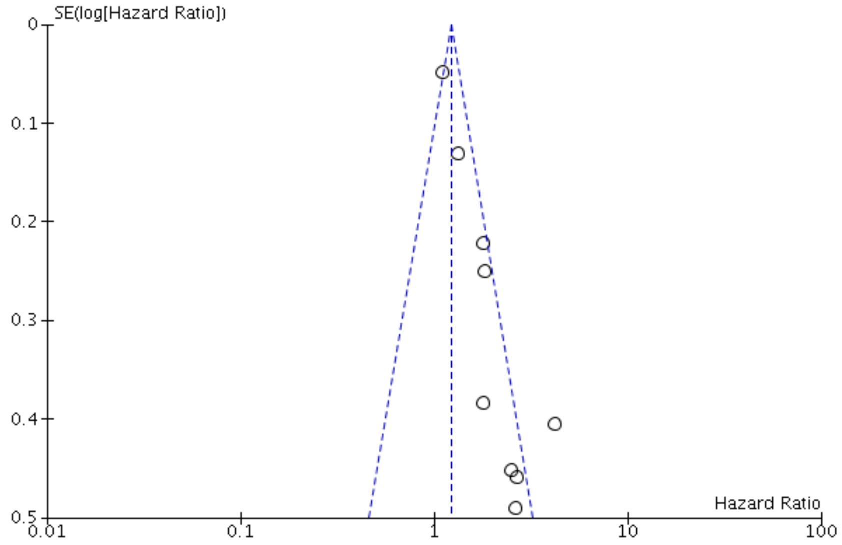

Funnel plot 2

The association between sleep disordered breathing and risk of cardiovascular diseases (Odds ratios) in an unselected general population

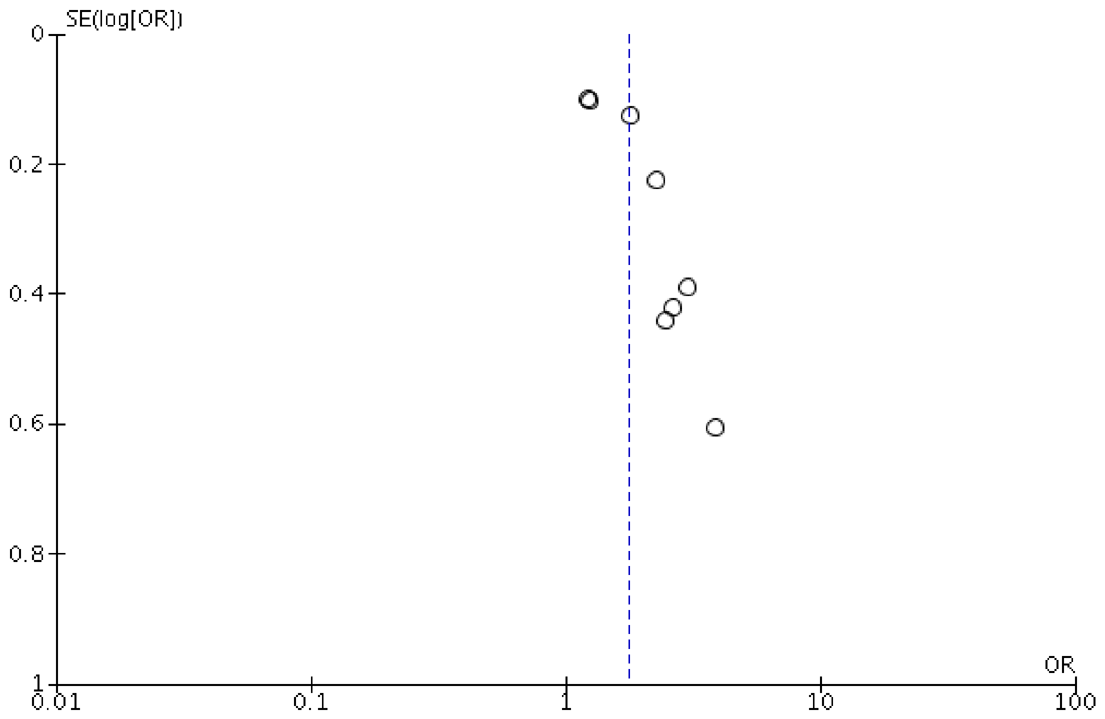

Funnelplot 3

The association between sleep disordered breathing and risk of cardiovascular diseases (Hazard ratios) in a population at high risk of sleep disordered breathing.

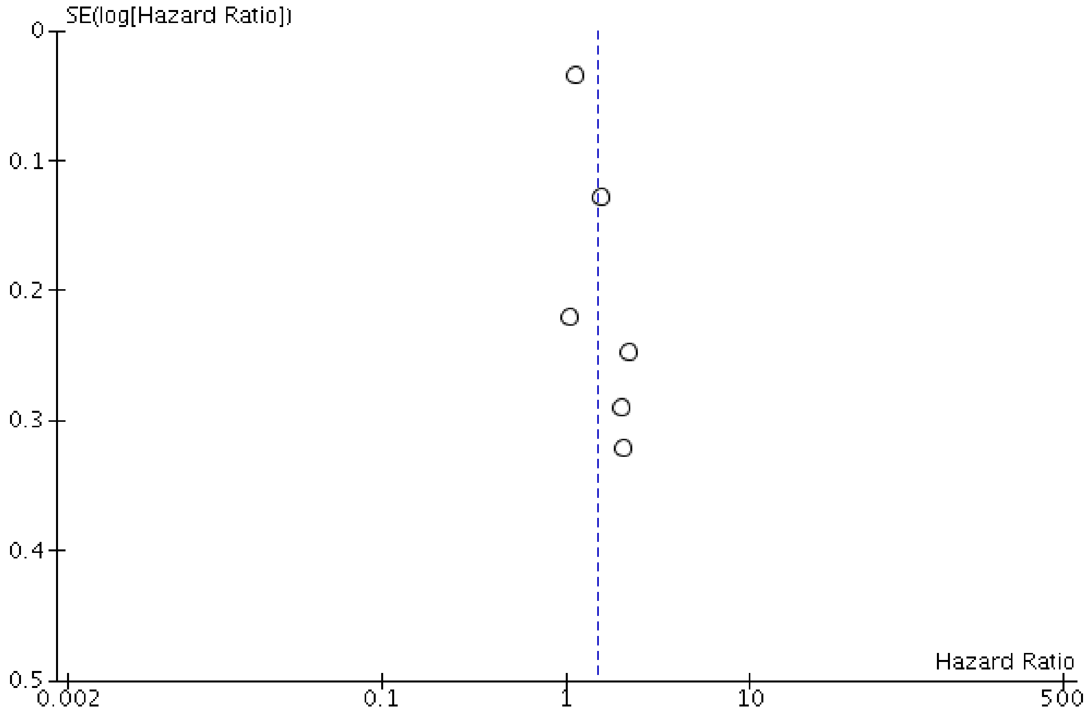

Supplement: Supplementary file 1 — Supplementary Information [file 41533_2022_307_MOESM1_ESM.pdf]
